# Aortic Stiffness Can be Predicted From Different eGFR Formulas With Long Follow-Up in the Malmö Diet Cancer Study

**DOI:** 10.1177/00033197241232719

**Published:** 2024-02-09

**Authors:** Anders Christensson, Simon Lundgren, Madeleine Johansson, Peter M. Nilsson, Gunnar Engström, Agne Laucyte-Cibulskiene

**Affiliations:** 1 Department of Clinical Sciences, 5193 Lund University, Malmö, Sweden; 2 Department of Nephrology, Skane University Hospital, Malmö, Sweden; 3 Department of Cardiology, Skåne University Hospital, Malmö, Sweden; 4 Cardiovascular Epidemiology, Department of Clinical Sciences, 5193 Lund University, Malmö, Sweden

**Keywords:** aortic stiffness, kidney function, cystatin C, epidemiology, estimated glomerular filtration rate

## Abstract

We studied the impact of estimated glomerular filtration rate (eGFR) based on either creatinine or cystatin C, or in combination, on vascular aging (aortic stiffness) and central hemodynamics (central systolic blood pressure) in a Swedish urban population with median 17 years of follow-up. Participants (*n* = 5049) from the population-based Malmö Diet and Cancer Study that underwent baseline examination and later participated in the prospective cardiovascular arm were selected. Of these, 2064 with measured carotid-femoral pulse wave velocity (cfPWV) and central blood pressure at follow-up were enrolled. eGFR was calculated using cystatin C (eGFR_CYS_) and creatinine (eGFR_CR_) equations: Caucasian, Asian, pediatric, and adult cohorts (CAPA), the Lund-Malmö revised (LMrev), and the European Kidney Function Consortium (EKFC) equations. Lower adjusted eGFR_CR_, but not eGFR_CYS_, were independently associated with higher cfPWV (*P* < .001, respectively). eGFR <60 mL/min/1.73 m^2^ determined higher cfPWV except when using the EKFC equation. Conversely, CAPA/LMrev and CAPA/EKFC ratios were not associated with aortic stiffness. Lower eGFR_CR_ is associated with higher future aortic stiffness independently of age, sex, heart rate, mean blood pressure, body mass index, and antihypertensive treatment. The ratio of eGFR_CYS_ and eGFR_CR_ equations could not predict aortic stiffness at all.

## Introduction

Vascular aging has become an important research topic in recent decades since it may reflect our biological age and play an essential role in predicting cardiovascular risk^1,2^ and all-cause mortality.^2^ In some conditions chronological age does not correspond with biological age, for example, in chronic kidney disease (CKD). The bidirectional pathophysiologic mechanisms between the cardiovascular and renal systems are associated with accelerated arterial stiffening in CKD.^3–5^ Considering that arterial stiffness proceeds hypertension,^6^ targeting it during its early stages seems to be important to reduce cardiovascular risk, spare cognitive function,^7,8^ and prevent kidney function decline.^9^

CKD represents a wide variety of vascular remodeling, including atherosclerosis, arteriosclerosis (or arterial stiffness), tunica intima and media calcification, and microcirculatory rarefaction.^10–13^ It remains uncertain whether aortic stiffness causes kidney function loss or the opposite—changes in kidney microcirculation and, consequently, increased peripheral resistance resulting in increased stiffness.^10^ This report aims to address aortic stiffness as an outcome related to baseline kidney function (estimated glomerular filtration rate, eGFR). We also address the issue of inconsistent results when comparing cystatin C-based eGFR with creatinine-based eGFR, recently defined as selective glomerular hyperfiltration syndrome or shrunken pore syndrome,^14^ and its association with future aortic stiffness.

With this background, we hypothesize that lower baseline eGFR based on cystatin C, or a combination of creatinine and cystatin C, predicts aortic stiffness, measured as carotid-femoral pulse wave velocity (cfPWV), after a mean 17 years of follow-up in a Swedish urban population.

## Materials and Methods

### Study Design and Participants

During 1991–1996, the population-based prospective Malmö Diet and Cancer Study (MDCS)^15,16^ recruited residents of Malmö city in Sweden: 17 035 women (with a primary goal to analyze incident breast cancer) and 11 063 men (with a primary goal to explore incident prostate cancer), born 1923–1950 and 1923–1945, respectively.

**Figure 1.**
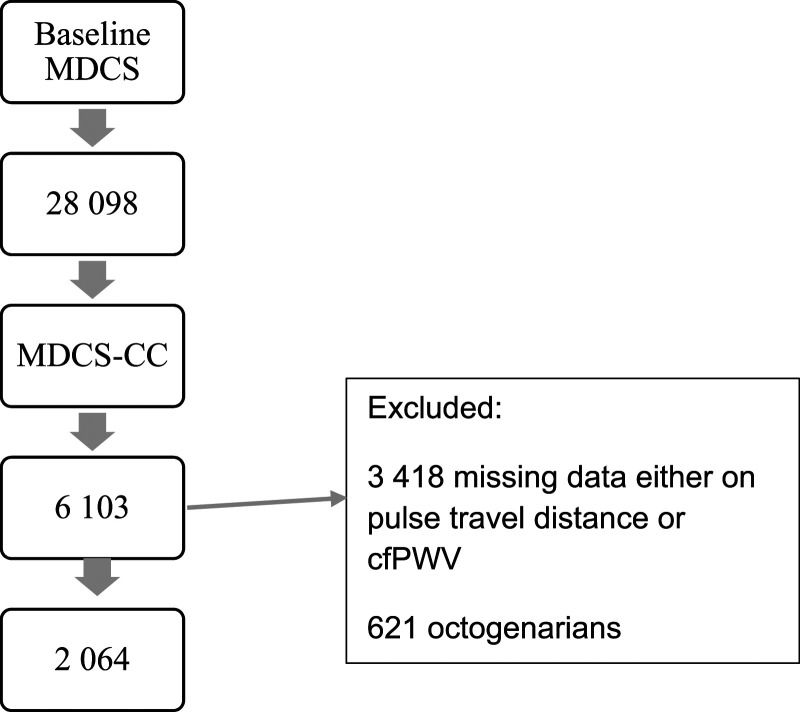
Flow chart of study participants. Abbreviations: MDCS, Malmo Diet Cancer study; MDCS-CC, Malmo Diet Cancer study cardiovascular cohort.

Between 2007 and 2012, some of these individuals (*n* = 6103) were randomly re-examined in the cardiovascular arm (MDCS‐CC).^17^ Out of 6103 participants at baseline (1994–1996), in all 2685 had both creatinine and cystatin C measured at baseline, as well as cfPWV measurements at the re-examination (2007–2012). This corresponds to a follow-up of 17 years (IQR 2.37, range 16–21 years). A total of 621 were octogenarians at the follow-up and therefore excluded from the study (see the flow chart in Figure 1).

### Measurements

#### Demographics

Diabetes mellitus was determined at baseline by a fasting blood glucose level ≥6.1 mmol/L, self-reported diabetes diagnosis, register-based prevalent diabetes diagnoses if available, or use of antidiabetic treatment. Hypertension was defined at baseline examination as use of antihypertensives, a previous diagnosis of hypertension, or a systolic blood pressure (SBP) ≥140 mmHg, and a diastolic blood pressure (DBP) ≥90 mmHg.

SBP and DBP at baseline were measured manually on the right overarm by trained nurses using a mercury-column sphygmomanometer twice in the supine position after 10 min of rest. Average blood pressure from two readings was computed. Mean arterial pressure (MAP) was calculated using the formula SBP+2DBP/3 (mmHg).

#### Biochemistry at Baseline

Fasting blood samples were collected and then frozen (−80°C) in a biobank. Following kidney function biomarkers were measured: plasma creatinine (μmol/L) (Jaffé method)^18^; plasma cystatin C (mg/L) (particle-enhanced immunonephelometric assay [N Latex Cystatin; Dade Behring, Deerfield, IL, USA]).^19^ Since cystatin C was analyzed before 2010 when the worldwide calibrator was introduced, we had to standardize the values by using methods as described previously.^20^

#### Central Hemodynamic and Measurement of cfPWV at Follow-Up

Central systolic blood pressure (cSBP) and the cfPWV were examined after a mean follow-up of 17 years (IQR 2.34) by using the applanation tonometry (SphygmoCor, AtCOR Medical, Sydney, Australia) that utilizes the foot-to-foot method to define the transit time through intersecting tangents algorithm. The heart rate was monitored using simple three-lead electrocardiography. Peripheral blood pressure (pSBP and pDBP) was measured after 10 min of supine rest with an automatic device (OMRON M5-1 IntelliSense, Kyoto, Japan).

First, the pulse wave was recorded on the pulse site of the carotid artery, and then on the pulse site of the femoral artery, simultaneous ECG registration was performed. Finally, the distance was measured by using a tape from the measuring point on the carotid artery (CA)—to the suprasternal notch (SSN)—then to the umbilicus (UMB)—and finally to the measuring point at the femoral artery (FA) site minus the suprasternal notch to the measuring point at the carotid artery:

Distance=(SSN−UMB−FA)−(SSN−CA)

Three cfPWV measurements were performed for each individual, and the average cfPWV value was calculated.

Since the CA-FA distance multiplied by 0.8 has the best agreement with real traveled aortic path length measured by using magnetic resonance imaging,^21^ we applied a conversion factor to calculate standardized values of cfPWV in our population:

$$\text{cfPWV}={\text{cfPWV}}_{\text{measured}}\times 1.083$$

A conversion factor “1.083” is based on MRI refrence values for pulse travel distance validated by Huybrechts et al.^21^

#### Estimation of Kidney Function at Baseline

Kidney function was determined by using creatinine and cystatin C-based eGFR formulas: cystatin C eGFR equation based on Caucasian, Asian, pediatric, and adult cohorts (CAPA)^22^; the Lund-Malmö revised creatinine-based eGFR equation (LMrev)^23^; and the European Kidney Function Consortium (EKFC) equation for creatinine.^24^ LMrev equation has been validated in a Swedish population^25^ and is superior to other creatinine-based equations, such as the Modification of Diet in Renal Disease (MDRD) study and Chronic Kidney Disease Epidemiology Collaboration (CKD-EPI).

To account for different cystatin C and creatinine filtration, previously described by Grubb^26^ and recently named selective glomerular hypofiltration syndromes,^14^ we calculated the ratio between CAPA and LMrev, and CAPA and EKFC.

### Statistical Analysis

For continuous variables, mean and standard deviations (SDs) were used; for discrete variables, medians with interquartile ratio (IQR) were used; and for categorical variables, numbers and percentages were used. Before deciding which test to use (mean comparison or nonparametric), F-test was performed to test the equality of two populations for normally distributed continuous data. For not normally distributed data, nonparametric tests were applied. Spearman´s correlation test was carried out to define the correlations among variables if at least one was not normally distributed. Adjusted *P-*values (Holm–Bonferroni method) were used to counteract the problem of multiple comparisons. eGFR was analyzed both as a continuous variable and as a categorical variable. We divided eGFR into groups: eGFR below <60, 60–90, and >90 mL/min/1.73 m.^2^

A generalized linear regression model (univariate and multivariate) was used for analyzing the relationship between eGFR (independent variable) and aortic stiffness (c-fPWV; dependent variable). We put all significant factors from univariate analysis into one multivariable model. After stepwise model selection adjustment for confounders such as age, sex, MAP, heart rate (HR), body mass index (BMI), and antihypertensive treatment available for 819 participants, creatinine-based eGFR remained relevant in predicting cfPWV. We used variation inflation factor (VIF) to address multicollinearity; variables excluded when sqrtVIF >2. The association between aortic stiffness and discrepancy in cystatin C/creatinine eGFR ratio was also analyzed.

Rcrmdr version R 3.6.2 GUI 1.70 El Capitan build macOS (McMaster University, Hamilton, Ontario, Canada) was used for statistical analysis. The significance level was defined by two-sided *P* < .05.

## Results

### General Characteristics of the Study Population

The general demographic characteristics are highlighted in Table 1. The data on excluded individuals is presented in Supplemental Table 1.

**Table 1.** Demographic and Clinical Characteristics of Study Subjects. Means (SD) or proportions.

Variable	Total (*n* = 2064)	Women (*n* = 1286)	Men (*n* = 778)
*Baseline characteristics*			
Age, at baseline, years	53 (4)	53 (4)	53 (4)
Diabetes, yes	27 (1.6)	15 (2.3)	12 (1.2)
Hypertension, yes	558 (27.0)	241 (31.0)	317 (24.7)
Smoking status†:			
- Current	393 (19.4)	241 (19.0)	152 (20.0)
- Former	807 (39.8)	364 (28.7)	443 (48.2)
BMI, kg/m^2^	25.1 (3.5)	24.7 (3.7)	25.7 (3.2)
pSBP, mmHg	136 (17)	135 (17)	139 (17)
pDBP, mmHg	86 (9)	84 (9)	88 (9)
MAP, mmHg	103 (11)	101 (11)	104 (11)
Cystatin C, mg/L	1.11 (0.21)	1.12 (0.22)	1.10 (0.19)
Creatinine, µmol/L	83 (14)	78 (12)	91 (14)
CAPA, mL/min/1.73 m^2^	68 (15)	68 (15)	69 (14)
LMrev, mL/min/1.73 m^2^	80 (12)	85 (11)	74 (10)
EKFC, mL/min/1.73 m^2^	77 (13)	74 (12)	82 (11)
eGFR_CAPA-LMrev_, mL/min/1.73 m^2^	74 (11)	76 (11)	71 (10)
eGFR_CAPA-EKFC_ mL/min/1.73 m^2^	73 (12)	76 (11)	71 (11)
*Follow-up measurements*			
Age, at follow-up, years	70 (4)	70 (4)	71 (4)
Smoking status†:			
- Current	151 (7.3)	93 (7.2)	58 (7.5)
- Former	979 (47.4)	552 (42.9)	427 (54.9)
Lipid-lowering treatment†, at follow-up, yes	31 (3.8)	17 (7.9)	14 (2.3)
Anti-HT treatment‡, at follow-up, yes	223 (27.2)	85 (22.8)	138 (22.8)
cfPWV, m/s	10.95 (2.45)	10.74 (2.26)	11.29 (2.70)
HR, bpm	67 (11)	68 (10)	65 (11)
pSBP, mmHg	134 (17)	134 (17)	135 (16)
pDBP, mmHg	76 (9)	75 (9)	77 (9)
cSBP, mmHg	121 (17)	121 (17)	121 (15)
cDBP, mmHg	75 (9 (	74 (9)	76 (9)
cPP, mmHg	47 (12)	47 (12)	45 (10)
MAP, mmHg	103 (11)	101 (11)	104 (11)

Data expressed as mean (± SD/IQR) or number (%).

Data available † for 1269 women and 761 men; ‡ for 604 women and 215 men.

Abbreviations: Anti-HT, antihypertensive treatment; WC, waist circumference; WHR, waist-to-hip ratio; pSBP, peripheral systolic blood pressure; pDBP, peripheral diastolic blood pressure; cSBP, central systolic blood pressure; cDBP, central diastolic blood pressure; MAP, mean arterial pressure; cPP, central pulse pressure; HR, heart rate; cfPWV, carotid-femoral pulse wave velocity; CAPA, cystatin C eGFR equation based on Caucasian, Asian, pediatric, and adult cohorts; LMrev, the Lund-Malmö revised creatinine-based eGFR equation; EKFC, European Kidney Function Consortium equation; eGFR_CAPA-LMrev_, average eGFR calculated from CAPA and LMrev; eGFR_CAPA-EKFC_, average eGFR calculated from CAPA and EKFC.

### Kidney Function and Arterial Stiffness

**Figure 2.**
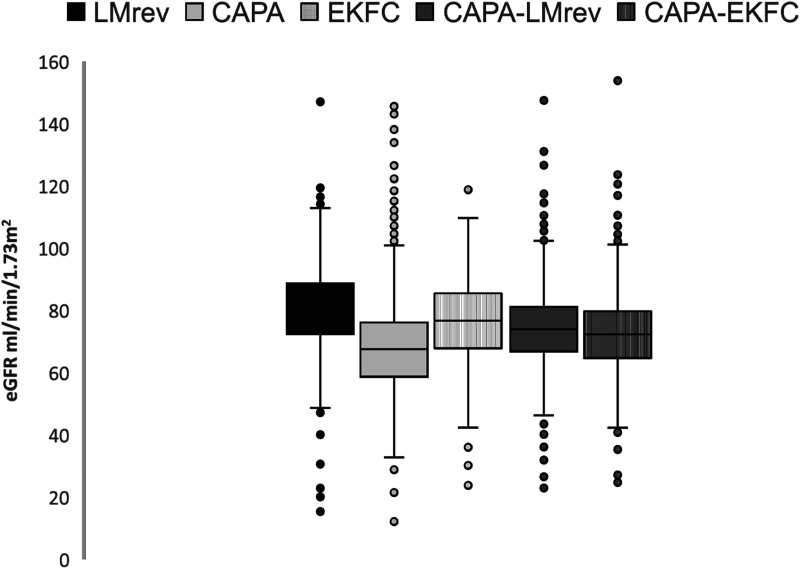
Distribution of baseline estimated glomerular filtration rate in the study subjects. Abbreviations: CAPA, cystatin C eGFR equation based on Caucasian, Asian, pediatric, and adult cohorts; LMrev, the Lund-Malmö revised creatinine-based eGFR equation; EKFC, European Kidney Function Consortium equation; CAPA-LMrev, average eGFR calculated from CAPA and LMrev; CAPA-EKFC, average eGFR calculated from CAPA and EKFC.

**Figure 3.**
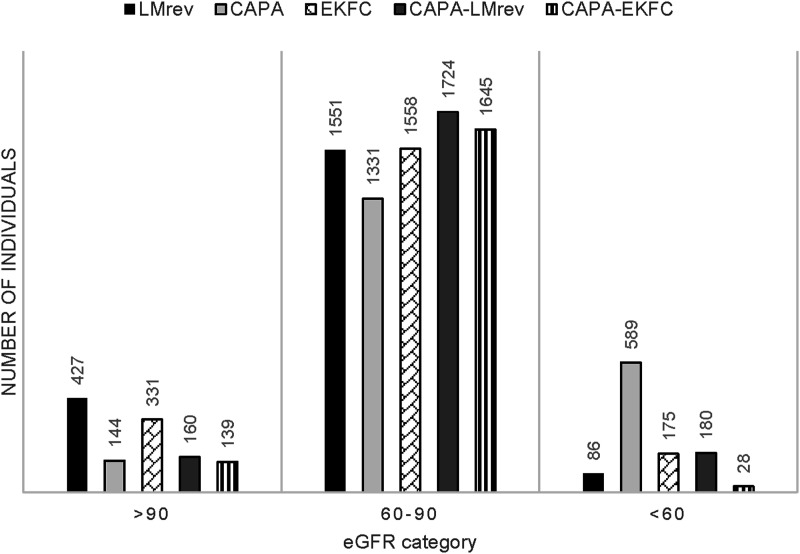
Classification of kidney function by different equations. Abbreviations: CAPA, cystatin C eGFR equation based on Caucasian, Asian, pediatric, and adult cohorts; LMrev, the Lund-Malmö revised creatinine-based eGFR equation; EKFC, European Kidney Function Consortium equation; CAPA-LMrev, average eGFR calculated from CAPA and LMrev; CAPA-EKFC, average eGFR calculated from CAPA and EKFC.

Kidney function at the inclusion to the study, calculated by five eGFR equations, is visualized in Figure 2. Figure 3 depicts how diverse eGFR equations classify kidney function among individuals studied.

We observed that lower baseline eGFR is associated with higher predicted cfPWV at follow-up (Table 2). Only creatinine-based equations performed well in identifying this relationship. Additionally, creatinine-based EKFC equation showed a very weak association with central systolic blood pressure (Table 2) that disappeared after multiple adjustments.

**Table 2.** Association Between Kidney Function and Carotid-Femoral Pulse Wave Velocity as Well as Central Systolic Blood Pressure.

Dependent variable	Ln (cfPWV)			cSBP		
	Univariate			Univariate		
	*ß*	*95% CI*	*P*	*ß*	*95% CI*	*P*
Age, years	0.016	0.01–0.02	**<0.001**	1.00	1.00–1.01	**<0.001**
Δ Age, years	0.003	−0.00–0.01	0.287	−0.53	−1.00–(-)0.05	**0.029**
Sex, female	−0.04	−0.06–(-)0.03	**<0.001**	0.58	−0.89–2.06	0.440
Diabetes, yes	0.13	0.05–0.21	**<0.001**	0.32	−6.03–6.67	0.921
Hypertension, yes	0.08	0.06–0.10	**<0.001**	6.23	4.65–7.83	**<0.001**
Antihypertensives, yes	0.08	0.04–0.10	**<0.001**	5.45	2.89–8.01	**<0.001**
Lipid-lowering drugs, yes	−0.01	−0.08–0.07	0.856	5.13	−1.18–11.43	0.111
BMI, kg/m^2^	0.01	0.08e-1–0.01	**<0.001**	0.28	0.08–0.49	**0.007**
MAP, mmHg	0.01	0.06e-1–0.07e-1	**<0.001**	1.33	1.30–1.35	**<0.001**
HR, bpm	0.04e-1	0.04e-1–0.05e-1	**<0.001**	−0.01	−0.07–0.06	0.883
CAPA, mL/min/1.73 m^2^	−0.01e-1	−0.02e-1–(-)0.03e-2	**0.003**	−0.02	−0.07–0.03	0.335
LMrev, mL/min/1.73 m^2^	−0.02e-1	−0.01e-1–(-)0.03e-1	**<0.001**	−0.04	−0.10–0.02	0.243
EKFC, mL/min/1.73 m^2^	−0.01e-1	−0.02e-1–(-)0.03e-2	**0.010**	−0.10	−0.12–(-)0.01	**0.024**
eGFR_CAPA-LMrev_, mL/min/1.73 m^2^	−0.02e-1	−0.01e-1–(-)0.03e-1	**<0.001**	−0.04	−0.10–0.02	0.217
eGFR_CAPA-EKFC_, mL/min/1.73 m^2^	−0.01e-1	−0.02e-1–(-)0.01e-1	**<0.001**	−0.06	−0.12–0.01	0.074

Generalized linear regression (identity link) model with logarithmed cfPWV and central systolic blood pressure (cSBP) as dependent variables.

Abbreviations: Δ Age, difference between age at follow-up and at the baseline; BMI, body mass index; HR, heart rate; MAP, mean arterial pressure; CAPA, cystatin C eGFR equation based on Caucasian, Asian, pediatric, and adult cohorts; LMrev, the Lund-Malmö revised creatinine-based eGFR equation; EKFC, European Kidney Function Consortium equation; eGFR_CAPA-LMrev_, average eGFR calculated from CAPA and LMrev; eGFR_CAPA-EKFC_, average eGFR calculated from CAPA and EKFC.

After stepwise model selection, LMrev and EKFC equations adjusted for age, sex, MAP, heart rate, antihypertensive medications, and body mass index showed the strongest significant relationship with cfPWV (Table 3). Neither Cystatin C nor eGFR average eGFR from a combination of cystatin C and creatinine were predictors of cfPWV.

**Table 3.** Stepwise Model Selection of Variables Independently Associated With Carotid-Femoral Pulse Wave Velocity.

Dependent variable	Ln (cfPWV)		
	Multivariate		
	*R* ^*2*^	*Mean Square*	*P*
Model 1	0.209	1.219	**<0.001**
Model 2	0.216	1.050	**<0.001**
Model 3a	0.222	0.926	**<0.001**
Model 3b	0.227	0.944	**<0.001**

Stepwise linear model selection for 819 subjects that had data on use of antihypertensive treatment, ln(cfPWV) as dependent variable. Models with CAPA equation failed at predicting cfPWV.

Model 1: adjusted for age, sex, MAP, heart rate, and antihypertensive medication;

Model 2: Model 1 + BMI;

Model 3a: Model 2 + LMrev;

Model 3b: Model 2 + EKFC.

Abbreviations: ln(cfPWV), natural logarithm of carotid-femoral pulse wave velocity; BMI, body mass index; CAPA, cystatin C eGFR equation based on Caucasian, Asian, pediatric, and adult cohorts; LMrev, the Lund-Malmö revised creatinine-based eGFR equation; EKFC, European Kidney Function Consortium equation; MAP, mean arterial pressure.

**Figure 4.**
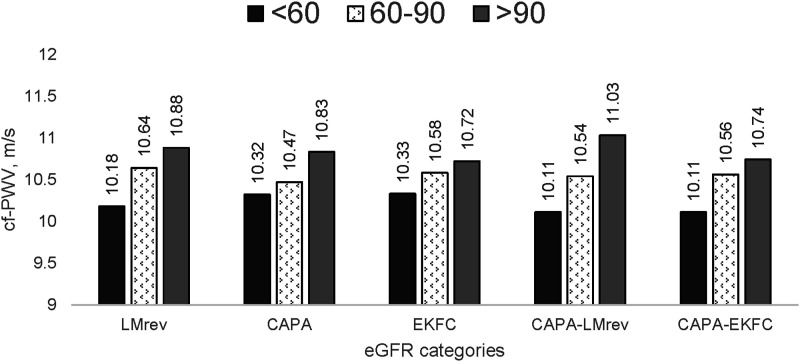
Carotid-femoral pulse wave velocity in categories of kidney function. Abbreviations: eGFR, estimated glomerular filtration rate; eGFR categories, estimated glomerular filtration rate divided into groups: below 60 mL/min/1.73 m^2^, between 60 and 90 mL/min/1.73 m^2^, and above 90 mL/min/1.73 m^2^; CAPA, cystatin C eGFR equation based on Caucasian, Asian, pediatric, and adult cohorts; LMrev, the Lund-Malmö revised creatinine-based eGFR equation; EKFC, European Kidney Function Consortium equation; CAPA-LMrev, average eGFR calculated from CAPA and LMrev; CAPA-EKFC, average eGFR calculated from CAPA and EKFC; cfPWV, standardized carotid-femoral pulse wave velocity values. *P <0.05; **P < .001.

Interestingly, when comparing cfPWV across eGFR categories (Figure 4), unlike the remaining four equations, the EKFC equation did not identify the variation of cfPWV (*P* = .102) among different eGFR levels. Of note, there were only 28 individuals classified as eGFR <60 mL/min/1.73 m^2^ according to the CAPA-EKFC equation.

Additionally, neither the ratio of CAPA/LMrev nor CAPA/EKFC was related to aortic stiffness in the whole cohort (Supplemental Figure 1).

## Discussion

Based on our observational findings, creatinine-based eGFR equations for renal function can predict aortic stiffness measured after a mean of 17 years in an urban, middle-aged Swedish population. Lower kidney function is thus independently associated with higher aortic stiffness in the future. This relationship is independent of age, sex, BMI, mean blood pressure, heart rate, and antihypertensive treatment. However, central blood pressure seems to be affected by factors other than historical kidney function. The discrepancy between cystatin C and creatinine-based equations was not associated with arterial stiffness in the whole cohort, suggesting different pathophysiological mechanisms responsible for large artery vascular aging and kidney aging.

Increased PWV, a marker of arterial stiffness, is one of the essential characteristics of vascular aging, reflecting arteriosclerosis.^13^ This study found that the *lower* the baseline creatinine-based eGFR, the *higher* cfPWV after a mean of 17 years of follow-up. This is the first report trying to link baseline kidney function to future aortic stiffness after such a long observational period in a population-based cohort. Previously published data from populations of comparable age and sex reported that in 482 individuals participating in the Maine Syracuse Longitudinal Study (MSLS),^26^ kidney function decline over 4–5 years was associated with increased cfPWV. In contrast, a study based on HIPPOCRATES (Hypertension: Interaction and Prevalence of POlymorphisms related to Cardiovascular Risk and the Association to Treatment Efficacy Study) cohort^27^ reported that in 222 subjects the baseline eGFR (CKD-EPI based on creatinine) could not predict annual cfPWV change during a follow-up of 5.6 years. These authors used creatinine-based equations: MDRD and CKD-EPI. We used eGFR equations, for example, LMrev, that are validated and adapted to the Swedish population.^23,25^ Furthermore, combining eGFR formulas from creatinine and cystatin C values—that is, calculating the average eGFR—helps to achieve more precise kidney function assessment. ^28^

Almost a decade ago, our colleagues^29^ published a paper analyzing non-hemodynamical determinants of arterial stiffness in the same cohort. They found that cross-sectionally eGFR—calculated using CKD-EPI, based on both cystatin C and creatinine (adjusted for age, MAP, and heart rate)—was associated with cfPWV. However, the significance disappeared after additional adjustments for BMI, current smoking, and medication. The main issues with this first report are noncalibrated cystatin C values that underestimated kidney function, adjusting for lipid-lowering drugs unrelated to cfPWV according to univariate analysis, and not considering different time periods between measurements in individuals. In our analysis, we could address all these previous pitfalls and reveal the previously unidentified relationship.

Some of the research up till now has observed a cross-sectional link between cystatin C and PWV^30–32^ or atherosclerosis^33^ in patients with no or mild CKD, yet lacking data on follow-up. Though cystatin C gene variant rs13038305 failed to show any causal relationship with cardiovascular disease and mortality,^34,35^ we believe that altered kidney filtration for cystatin C might be the key player in cardiovascular remodeling. The relation between heart and kidney, represented by the cardiorenal syndrome, may be caused by common vascular changes giving rise to different elimination patterns for small- and middle-sized molecules described in the so-called selective glomerular hypofiltration syndromes^14^ represented by a cystatin C/creatinine-based eGFR ratio <0.7. Observed clearance changes of molecules with different molecular masses have been associated with an accumulation of middle-sized atherosclerosis-promoting proteins, but not with small molecules like creatinine.^36^ We could not find an association between low cystatin C/creatinine-based eGFR ratio and future cfPWV that could be similar to previously reported relationship with the cardiorenal syndrome.^37^ One explanation is the lack of cross-sectional data and the limited knowledge about events that could have affected the course of vascular aging. The complex mechanism in micro- and macrovascular remodeling^38^ inconsistent with those possibly related to selective hypofiltration may be the other reliable explanation for this lack of association. However, further studies should focus on how microvascular changes affect sieving for molecules with greater masses in kidneys and if they are related to renal resistance.^39^

The strength of this study is standardizing of cfPWV on the actual aortic path length based on MRI studies, the prospective evaluation of aortic stiffness, and use of eGFR equations adapted to the studied population. Adding cystatin C analysis to routine creatinine analysis is a cost-effective method that improves the diagnosis of CKD and risk prediction.

The limitation is that we did not have interim measurements either of kidney function or aortic stiffness that could depict the time slope of these variables. In addition, during these 17 years, many other risk factors could have influenced kidney function, in particular chronological age. Therefore, octogenarians were excluded from the analysis. However, we assume that subjects who agreed to participate in the re-examination were likely not affected by a high disease burden (health selected) since these cardiovascular examinations were rather extensive and time-consuming.^17,40^ Another limitation is that only office blood pressure (BP) was used at baseline screening and not 24-h ambulatory blood pressure measurement, a more precise measure of hemodynamic function and BP.

In summary, we identified that lower kidney function based on creatinine and cystatin C could predict future aortic stiffness in a middle-aged Swedish population, independent of mean arterial pressure. The phenomenon of lower eGFRcystatin C/eGFRcreatinine ratio could not be shown in this study. Further studies are warranted to analyze the relationship between selective hypofiltration syndromes and vascular aging, preferably in a cross-sectional manner.

## Supplemental Material

Supplemental Material - Aortic Stiffness Can be Predicted From Different eGFR Formulas With Long Follow-Up in the Malmö Diet Cancer Study Supplemental Material for Aortic Stiffness Can be Predicted From Different eGFR Formulas With Long Follow-Up in the Malmö Diet Cancer Study by Anders Christensson, MD, PhD, Simon Lundgren, MD, Madeleine Johansson, MD, PhD, Peter M. Nilsson, MD, PhD, Gunnar Engström, PhD, and Agne Laucyte-Cibulskiene, MD, PhD in Angiology.

## Data Availability

The datasets generated and/or analyzed during the current study, after deidentification (text, tables, figures, and appendices), could be extracted by sending application to MDC steering committee on reasonable request. Proposals should be directed to anders.dahlin@med.lu.se. To gain access, data requestors will need to sign a data access agreement.
