# Supplementary material for: Aortic Stiffness Can be Predicted From Different eGFR Formulas With Long Follow-Up in the Malmö Diet Cancer Study
Source: Angiology. 2024 Feb 9;76(6):563–71. doi: 10.1177/00033197241232719 (PMC12222838; doi:10.1177/00033197241232719)
Supplement: Supplemental Material - Aortic Stiffness Can be Predicted From Different eGFR Formulas With Long Follow-Up in the Malmö Diet Cancer Study [file sj-pdf-1-ang-10.1177_00033197241232719.pdf]

Supplemental Table 1. Comparison of baseline clinical characteristics of included and excluded individuals.

**Variable**	**Total (n=2064)**	**Excluded (n=621)**	***P*-value**
Age, at baseline, years	53 (4)	63 (2)	<0.001
Age, at follow-up, years	70 (4)	80 (2)	<0.001
Sex, women	1286 (62.3)	373 (60.1)	0.313
Diabetes, yes	27 (1.6)	7 (1.4)	0.840
Hypertension, yes	558 (27.0)	244 (39.3)	<0.001
Smoking status:			
- Current	393 (19.4)	67 (10.9)	<0.001
- Former	807 (39.8)	246 (40.0)	0.818
Lipid-lowering treatment, at follow-up, yes†	31 (3.8)	20 (7.3)	<0.001
AntiHT treatment, at follow-up, yes‡	223 (27.2)	111 (40.7)	<0.001

Data expressed as Mean (± SD/IQR), or number (%).

Abbreviations: AntiHT, antihypertensive treatment.

Data available: † for 2030 individuals included and 306 individuals excluded from the study; ‡ for 819 inviduals included and 273 individuals excluded from the study.

Supplemental Figure 1. Correlation between cfPWV and kidney function, represented as the quotient between eGFRcys and eGFRcr.

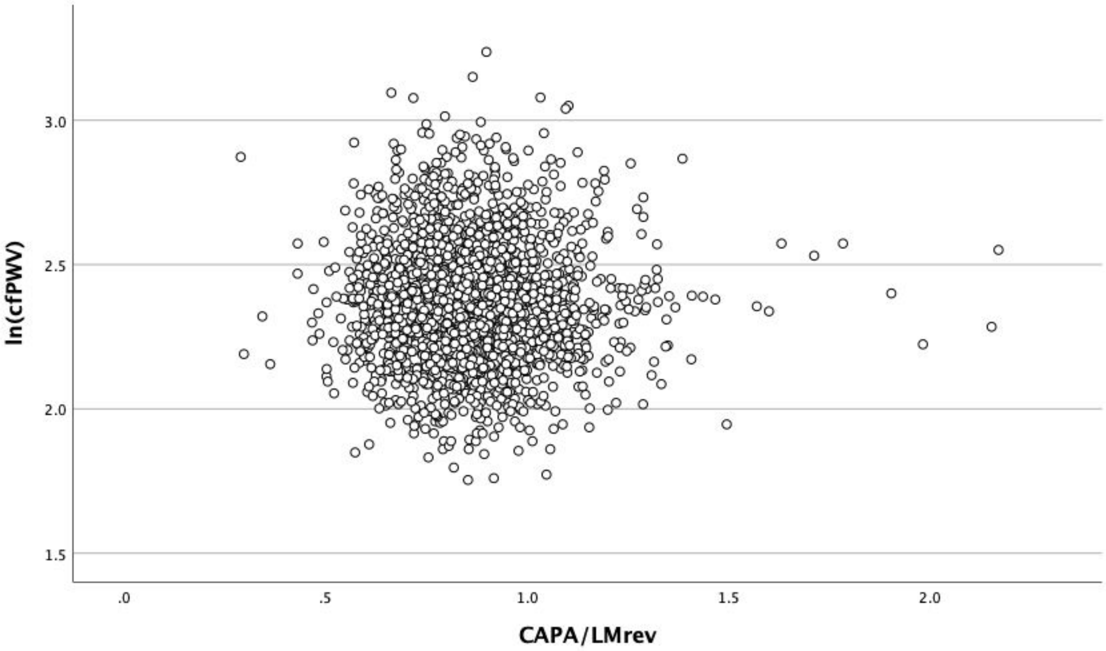

r = 0.02, p = 0.50

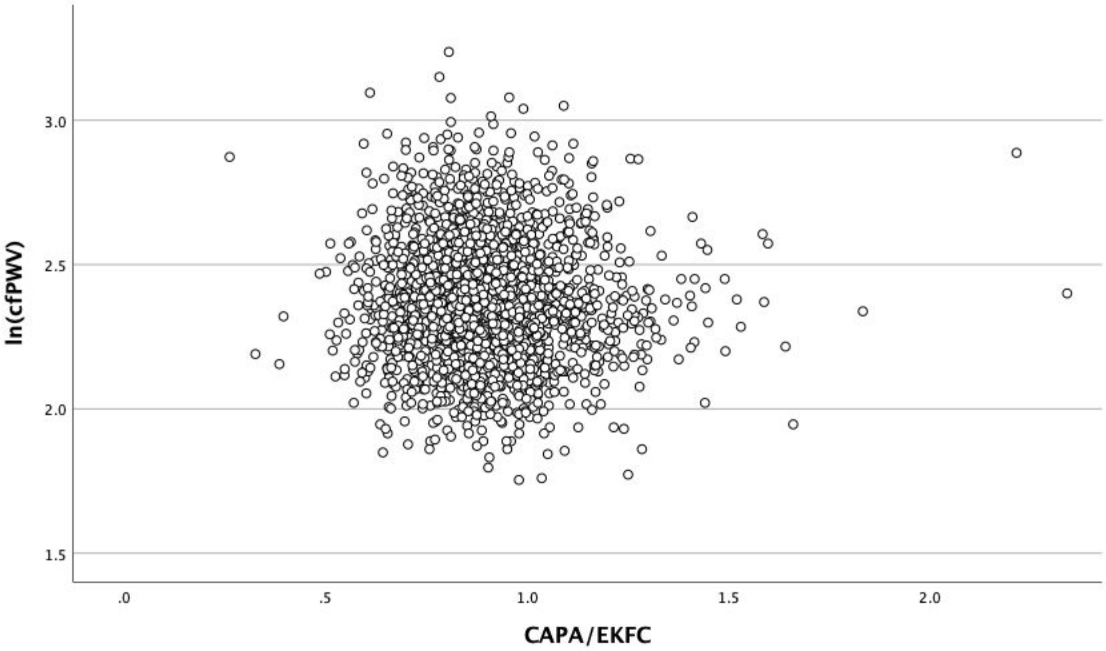

r = -0.03, p = 0.26

Abbreviations: ln(cfPWV), natural logarithm of carotid-femoral pulse wave velocity; CAPA, cystatin C eGFR equation based on Caucasian, Asian, pediatric, and adult cohorts; LMrev, the Lund-Malmö revised creatinine based eGFR equation; EKFC, European Kidney Function Consortium equation; CAPA/LMrev, ratio between CAPA and LMrev; CAPA/EKFC, ratio between CAPA and EKFC.

http://mc.manuscriptcentral.com/ang
